# Endometrial SUSD2^+^ Mesenchymal Stem/Stromal Cells in Tissue Engineering: Advances in Novel Cellular Constructs for Pelvic Organ Prolapse

**DOI:** 10.3390/jpm11090840

**Published:** 2021-08-26

**Authors:** David M. Z. B. Hennes, Anna Rosamilia, Jerome A. Werkmeister, Caroline E. Gargett, Shayanti Mukherjee

**Affiliations:** 1 The Ritchie Centre, Hudson Institute of Medical Research, Clayton, VIC 3168, Australia; AnnaRosamilia@urogyn.com.au (A.R.); jerome.werkmeister@hudson.org.au (J.A.W.); caroline.gargett@monash.edu (C.E.G.); 2 Department of Obstetrics and Gynaecology, Monash University, Clayton, VIC 3168, Australia; 3 Pelvic Floor Disorders Unit, Monash Health, Clayton, VIC 3168, Australia

**Keywords:** endometrium, menstrual blood, MSC, perivascular MSC, cell culture, tissue engineering, bioscaffolds, biomaterials, nanofiber, electrospinning

## Abstract

Cellular therapy is an emerging field in clinical and personalised medicine. Many adult mesenchymal stem/progenitor cells (MSC) or pluripotent derivatives are being assessed simultaneously in preclinical trials for their potential treatment applications in chronic and degenerative human diseases. Endometrial mesenchymal stem/progenitor cells (eMSC) have been identified as clonogenic cells that exist in unique perivascular niches within the uterine endometrium. Compared with MSC isolated from other tissue sources, such as bone marrow and adipose tissue, eMSC can be extracted through less invasive methods of tissue sampling, and they exhibit improvements in potency, proliferative capacity, and control of culture-induced differentiation. In this review, we summarize the potential cell therapy and tissue engineering applications of eMSC in pelvic organ prolapse (POP), emphasising their ability to exert angiogenic and strong immunomodulatory responses that improve tissue integration of novel surgical constructs for POP and promote vaginal tissue healing.

## 1. Introduction

Pelvic organ prolapse (POP) is a common urogynaecological disorder that affects one in four women across all age groups, or over 50% of postmenopausal parous women with a history of vaginal birth [1,2]. POP is defined as the descent of the anterior and/or posterior vaginal wall, the apex of the vagina, or the vault, into or past the vaginal introitus, and presents significant yet hidden clinical burdens of disease with bowel, bladder, or sexual dysfunction that profoundly impact the quality of life of sufferers [3]. Up to 19% of women have a lifetime risk of undergoing reconstructive surgery for POP [4,5], with a 30–35% risk of reoperation due to recurrent anatomical failure [6], or adverse events associated with primary surgery [7].

In the recent past, urogynaecologists have implemented polypropylene (PP) mesh to augment POP surgery in an attempt to reduce the risk of anatomical failure. This mesh comprised synthetic, non-degradable macroporous monofilament fibres that caused serious adverse events in some women when implanted vaginally; including infection, retraction, exposure, and erosion [8]. The frequency and severity of these post-operative complications culminated in the Food and Drug Administration (FDA) issuing warnings against their use, and the subsequent prohibition of their use in transvaginal POP surgery in Australia, New Zealand, USA, and UK [9]. The adverse events associated with use of PP mesh in pelvic reconstructive surgery has been attributed to a lack of biomechanical compatibility of synthetic mesh with the unique dynamics of vaginal tissue, which in turn is associated with an exaggerated foreign body response that results in chronic fibrosis [10]. 

Thus, there is a critical need to provide novel surgical constructs that are not only safe and surgically efficacious, but also congruent with the native tissue, to maximise post-operative tissue healing and mesh integration. Tissue engineered surgical constructs consisting of endometrial mesenchymal stem cells (eMSC), combined with mesh, have demonstrated improved surgical outcomes in pre-clinical models of POP surgery, with a more favourable immune response and improved biomechanical properties of vaginal tissue [11]. This review will highlight key points in the trajectory of eMSC discovery, emphasising their potential benefit when combined with novel meshes in enhancing tissue integration and modulating inflammatory responses after mesh augmented pelvic reconstructive surgeries.

## 2. Pelvic Organ Prolapse

### 2.1. Aetiology

POP is a significant urogynaecological disorder that profoundly impacts the lives of millions of women worldwide due to consequent bladder, bowel, and sexual dysfunction [12]. Risk factors include multiple vaginal births (>3), difficult obstetric history, macrosomia, pregnancy, ageing, obesity, diabetes, hypertension, chronic straining, and coughing [13]. Other significant risk factors include the use of forceps, and other gynaecological procedures such as hysterectomy for other clinical indications [5,14,15].

### 2.2. Anatomy, Pathophysiology, and Biomechanics

**Figure 1:**
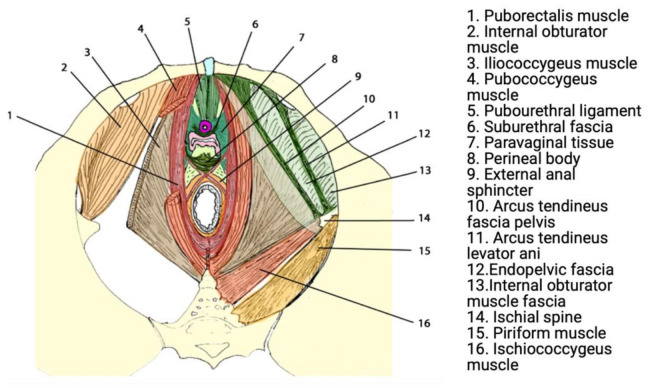
Functional anatomy of the pelvic floor. Reprinted with permission from Lamblin et al. (2016) [23]. Copyright Springer Nature, 2015.

Urogenital prolapse results from anatomic deficits in the ligamentous, fascial, and muscular structures that support the pelvic organs in sequence [16]. These structures have the crucial role of supporting abdominopelvic viscera through tonic contraction, passive resistance to increases in intra-pelvic/abdominal pressure (e.g., coughing), and crucially contribute to urinary and faecal continence. The most significant structure contributing to the pelvic floor is the levator ani muscle (LAM), which comprises three sling-shaped muscles known as the puborectalis, pubococcygeus, and iliococcygeus [17,18] (Figure 1). 

Vaginal birth results in stress to the vaginal wall and surrounding ligamentous, fascial, and muscular structures, beyond their critical stretch limit, resulting in tissue damage through non-elastic deformation [19]. During labour, pressure from strong uterine contractions and the transiting foetal head are directed to the LAM muscle, particularly the medial portion of the pubococcygeus. This results in an increased stretch ratio of up to 3.2, in contrast to a previous non-introitus stretch ratio of 2.17, which damages muscle fibres through non-elastic deformation [20,21,22]. 

### 2.3. Treatment of POP

Conservative treatment of POP incorporates non-operative approaches such as weight loss, avoiding exacerbating activities (lifting, constipation, and coughing), pelvic floor muscle training (PFMT), and various vaginal pessaries [24,25]. Failure of these treatments necessitates surgery in an estimated 20% of women, which includes native tissue repairs and/or mesh augmentation [4]. The operative approach is dependent on unique patient and pathological factors such as age, weight, physical activity level, severity of prolapse and symptoms, and expected durability of repair [25].

**Figure 2:**
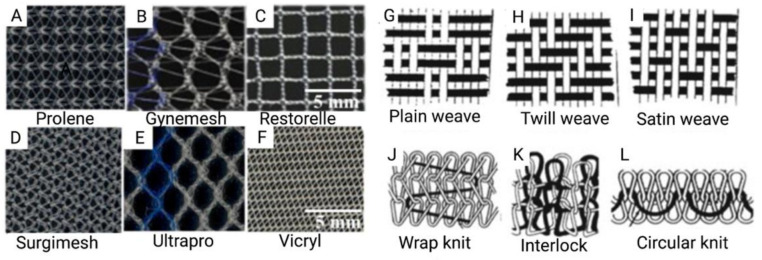
(**A**–**F**) Commercially available prosthetic meshes for POP; (**G**–**L**) Illustrations of mesh topography demonstrating different weave and knit types. Reprinted from Pott et al. (2012) [31]. Copyright: © 2021 by the authors.

In the past, pelvic reconstructive surgeons had to balance the higher risk of anatomical failure with native tissue repair with the risk of post-operative complications from mesh augmented repair [26]. Many surgeons chose to optimize the durability of the repair, and thus the increased use of synthetic non-degradable PP meshes for POP surgery followed [27,28,29,30] (Figure 2). 

### 2.4. Clinical Adversities

**Figure 3:**
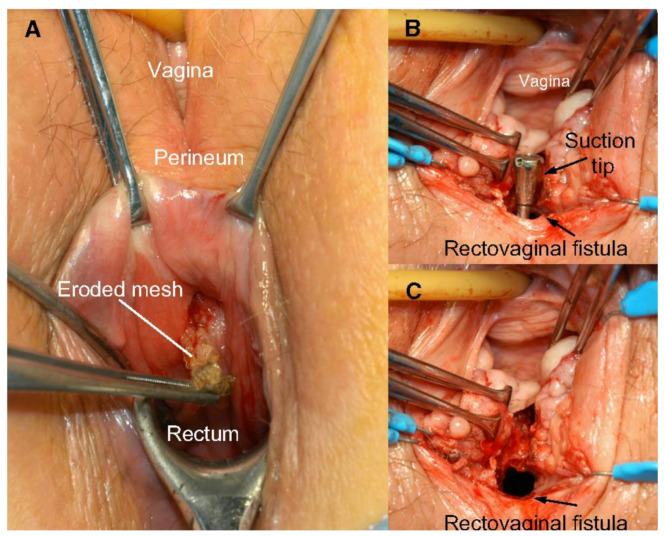
(**A**) Rectovaginal fistula formation secondary to mesh erosion through posterior vaginal wall; (**B**) rectal side of rectovaginal fistula after mesh removal; and (**C**) vaginal side of rectovaginal fistula after mesh removal. Reprinted with permission from Margulies et al. (2012) [34]. Copyright Elsevier, 2008.

**Figure 4:**
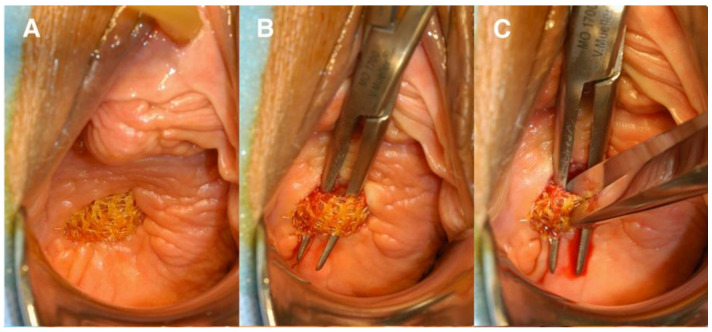
(**A**) Anterior vaginal wall with mesh exposure; (**B**) undermining the mesh; (**C**) transecting the mesh. Reprinted with permission from Margulies et al. (2012) [34]. Copyright Elsevier, 2008.

The use of transvaginal PP mesh resulted in post-operative complications including chronic pelvic and sexual pain, infection, extrusion, and erosion of mesh [32]. Mesh exposure occurs when the mesh extrudes from the vaginal mucosa, while mesh erosion occurs when mesh invades into other surrounding structures such as the bladder, rectum, or urethra [9] (Figure 3 and Figure 4). Even after optimized tertiary management of mesh complications, the impact of these adverse effects cause physical and emotional pain, in addition to the discomfort caused by original pelvic floor dysfunction. Feelings of anxiety, desperation, hopelessness, and abandonment are some detrimental emotional corollaries of adverse events that can impact a woman’s quality of life in the long-term [33]. 

The adverse events associated with synthetic mesh has been attributed to a number of factors. Firstly, an incompatibility of various mesh characteristics such as size, weight, stiffness, thickness, and porosity to comply with the unique dynamics of vaginal tissue [35]. Secondly, a suboptimal foreign body response resulting in increased matrix metalloproteinase (MMP) activity, which disrupts the integrity of the histological layers of the vagina through tissue degradation, particularly affecting the vaginal adventitia and smooth muscle fibres of the muscularis layer [36,37,38]. Transvaginal meshes used in vaginal repair should promote healthy tissue formation and remodelling while minimizing the host foreign body response. 

At present, there is a large void in the surgical management of vaginal prolapse which presents a need for novel bioengineered surgical constructs. New generations of mesh must consider the unique properties of the vaginal tissue they are designed to mimic in their nanostructure topography, porosity, and stiffness. The amalgamation of highly specialised disciplines such as tissue engineering, stem cell therapy and personalised medicine provide important approaches and tools to respond to this challenge. 

## 3. Mesenchymal Stem/Progenitor Cells 

The primary focus of cell-based therapies in the past has been adult multipotent mesenchymal stem or stromal cells (MSC). These cells have been pursued for their clonogenicity, proliferative capacity, differentiation to mesodermal lineages, secretion of angiogenic factors, and many other growth-promoting factors [39,40,41]. MSC provide therapeutic potential through their interaction with the both the innate and adaptive immune systems, by direct cell-cell contact and/or MSC secretion of immunosuppressive factors such as Indoleamine 2,3 deoxygenase (IDO), prostaglandin E2 (PGE2), nitric oxide (NO), human leukocyte antigen G5 (HLA-G5), IL-10, IL-6, and TGF-β [40]. This results in the inhibition of T and B cell proliferation, conversion of Th17 cells toward regulatory T cells (Tregs), and macrophage switching from M1 pro-inflammatory phenotype to anti-inflammatory M2 polarisation [42,43,44]. By acting in a paracrine manner, MSC possess the ability to promote endogenous cell proliferation, stimulate angiogenesis, and reduce fibrosis to effect repair of damaged host tissues [45]. 

MSC have typically been isolated from bone marrow through plastic adherence, and have also been extracted from other tissue including umbilical cord and unfractionated adipose tissue. However, MSC isolated from these sites have had limited clinical efficacy, due to their variable potency, lack of reproducibility in cell culture, and high rates of culture-induced spontaneous differentiation to fibroblasts [43]. For instance, the number of MSC in bone marrow significantly decreases with ageing, with an estimated tenfold loss for each decade of life [46]. Another major hurdle of MSC clinical translation has been the often painful and invasive techniques of tissue sampling involved in sourcing MSC, such as bone marrow aspiration or liposuction [47]. 

Human endometrial MSC (eMSC) have emerged as a novel source of therapeutic immunomodulatory cells isolated from an endometrial biopsy specimen, collected in an office-based procedure without anaesthesia, or harvested at the time of hysteroscopy and hysterectomy performed for other clinical indications [47]. The ease of sampling eMSC presents the opportunity to overcome one of the hurdles associated with clinical translation of bone marrow and adipose MSC. In their natural environment, eMSC are highly proliferative perivascular cells, functioning in generating vasculature to support rapid endometrial growth each menstrual cycle [39]. 

During the first 4–10 days of each menstrual cycle, 5–10 mm of new endometrial mucosa known as the functionalis grows from the basalis (0.5–1 mm thick) not shed during menstruation, into which an embryo subsequently implants [48]. If implantation does not occur, the vascularised and secretory endometrium sheds during days 1–4 of the subsequent menstrual cycle. Within 48 h of endometrial shedding, rapid repair/re-epithelialization of the endometrial surface occurs to cover the exposed basalis surface followed by regeneration of the functionalis layer [49]. This process repeats itself around 400 times across a woman’s reproductive life. Gene profiling has demonstrated that the lysed stroma of menstrual endometrium is enriched in genes involved in extracellular matrix (ECM) biosynthesis and degradation [50]. The ability for eMSC to promote the deposition of new ECM makes it an attractive source for technical applications in novel therapeutic POP constructs. 

eMSC were first identified in the perivascular stroma of mouse endometrium, as stromal label-retaining cells (LRCs) with about 13% located around blood vessels and 13% at the endometrial–myometrial junction [51]. Studies on human endometrium obtained from hysterectomy tissue demonstrated the existence of small numbers of stromal cells capable of forming colonies initiated by stromal stem/progenitor cells or colony-forming unit fibroblasts (CFU- F) [52], thereby indicating their pluripotent potential. Though a greater percentage of CFU-F was observed in proliferative endometrium under the influence of rising circulating oestrogen levels, they were also detected in the non-cycling atrophic endometrium from postmenopausal women and women on oral contraceptives [53,54]. 

**Figure 5:**
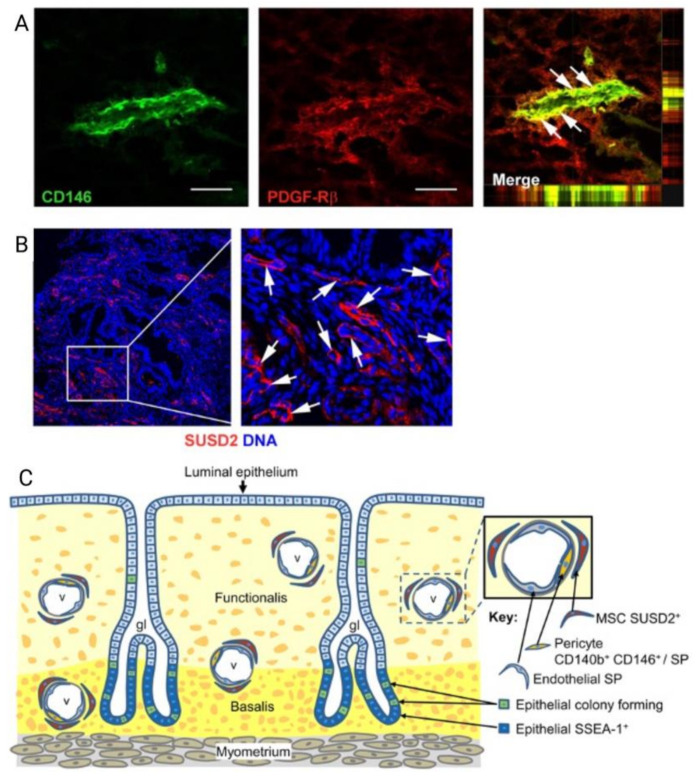
Localisation of human endometrial mesenchymal stem cells (eMSC). (**A**) Immunofluorescence images of human endometrium showing colocalization markers CD146 and platelet derived growth factor receptor beta (PDGF-Rβ, CD140b) to reveal the perivascular niche of eMSC (merge in the right of panel **A**); (**B**) perivascular Sushi Domain containing-2 (SUSD2) expression showing perivascular location (white arrows); and (**C**) Schematic showing the perivascular identity of co-expressing CD146 and PDGFRβ/CD140b and SUSD2^+^ eMSC in the endometrial basalis and functionalis layer, indicating eMSC will be shed into menstrual blood. Scale bar in (**A**) = 50 µm. Reprinted with permission from Gargett et al. (2010) [65]. Copyright Oxford University Press, 2010.

However, like all MSC, the challenges of eMSC lie in isolating them from the perivascular stroma, and limiting spontaneous differentiation to non-clonogenic fibroblasts during the culture expansion [55,56]. The identity of clonogenic eMSC was revealed by showing their enrichment in endometrial stromal cells, colocalizing two perivascular markers, CD140b (PDGFRb) and CD146 and their perivascular location (Figure 5) [55]. Gene profiling distinguished CD140b^+^CD146^+^ MSC within a perivascular niche in human endometrium from CD140b^+^CD146^−^ endometrial fibroblasts, which differentially expressed 762 other genes (Figure 5) [57]. The use of these markers allows prospective isolation and colocalization of eMSC, and supports the existence of eMSC within subpopulations of pericytes in human endometrium [58]. A single marker, W5C5 or Sushi Domain-containing 2 (SUSD2) is now used to purify and isolate rare perivascular eMSC from surrounding endometrial stromal cells [58]. SUSD2 enables prospective isolation of eMSC from freshly isolated cell suspensions using magnetic bead sorting, providing a more clonogenic population than obtained by flow cytometry sorting, which adversely affects cell viability [59,60]. 

A small molecule transforming growth factor-beta receptor (TGFRβ) inhibitor, A83-01, aids culture expansion of eMSC and other reproductive MSC, by maintaining them in the undifferentiated state promoting MSC proliferation, and preventing apoptosis and senescence [61,62]. Previous studies using functional assays and whole transcriptome sequencing have demonstrated that A83-01 promoted proliferation and increased cloning efficiency in premenopausal SUSD2 eMSC, providing insight into their biological activity [61,62,63]. The potency of A83-01 treated eMSC was also demonstrated by the increased expression of novel bone marrow MSC (bmMSC) potency genes *TWIST1*, *TWIST2*, *JAG1*, *LIFR*, and *SLIT2* in a novel assay of A83-01 treated MSC [62,64]. A83-01 treated eMSC are posited to improve tissue regeneration through the upregulation of pro-angiogenic factors and inhibition of anti-angiogenic factors, improved cell migratory capacity, promoting angiogenesis demonstrated by increased endothelial cell tube formation, and downregulation of fibrosis-associated gene transcription [62]. Furthermore, RNA sequencing studies have confirmed that eMSC secrete exosomes, potentially explaining their paracrine immunomodulatory effects [62]. A83-01 treated eMSC differentially expressed classic exosome markers, *CD63*, *CD9*, *CD81*, and *CD82*, confirming their paracrine immunomodulatory potential that could be exploited in regenerative or personalized medicine [62]. We are currently investigating whether or not eMSC produce exosomes distinguished from other types of MSC; however, data is insufficient to comment on this at present.

Similar to other MSC, eMSC exert strong immunomodulatory and anti-inflammatory effects primarily via paracrine cross-talk with cells of the innate and adaptive immune system, including macrophages, T cells, and NK cells. These effects may be harnessed to enhance the success of transvaginal mesh surgery reducing pathological foreign body responses, thereby promoting tissue integration. In addition, their perivascular identity makes them good candidates for regenerative medicine through the regulation of angiogenesis, inflammation, and fibrosis [11,66] (see Table 1). 

Given eMSC demonstrate angiogenic activity, concerns have been raised about their potential tumorigenicity and carcinogenicity. In an immunosuppressed xenograft rat model of brain stroke, menstrual blood stromal fibroblasts not only failed to stimulate, but also inhibited tumour growth and showed no evidence of tumour formation [77]. Similar anti-tumourigenic properties have not been demonstrated in eMSC to date, though no cancer due to culture expanded MSC has been diagnosed in pre-clinical or clinical studies [60]. Genomic studies have demonstrated that eMSC cultured in A83-01-containing medium may promote the stability of telomeres, through upregulated genes such as *TERC* (Telomerase RNA Component), *TERF1* and *2* (Telomeric Repeat Binding Factors 1 and 2), *TINF2* (TERF1 Interacting Nuclear Factor 2), *TERF2IP* (TERF2 Interacting Protein), *TNKS* (Tankyrase) and *POT1* (Protection of Telomere 1), which collaborate to regulate telomere length and protect cells from chromosomal damage [62]. Nonetheless, the potential for genomic instability needs to be further assessed through transcriptomics, karyotyping and in animal models. 

In addition to their enhanced therapeutic potential, another strength of eMSC is their retrieval through a relatively less invasive and cost-effective method of sampling that is not limited by the patient’s age, menopausal status, or medical comorbidities. Furthermore, by drawing upon the principles of personalised medicine, there is an exciting opportunity to use autologous eMSC derived from each patient to create a patient-specific tissue engineered cellular surgical construct. 

## 4. Engineering Novel Meshes with eMSC

Tissue engineering involves the creation of a tissue implant or substitute comprising a scaffold seeded with stem/progenitor cells, growth factors and/or drugs for restoring normal tissue function [38,39]. The biomaterial scaffold functions as physical support but also provides a three-dimensional nanostructured environment onto which therapeutic mesenchymal stem/progenitor cells may adhere, to accelerate and promote tissue regeneration and repair. Recently, it has been proposed that tissue engineering approaches combining autologous stem/progenitor cells with biomaterials fabrication could drastically improve the outcomes of pelvic reconstructive surgery [39,78]. 

### 4.1. eMSC Non-Degradable Tissue Engineered Mesh 

Our group has aimed to test the therapeutic potential of tissue engineering constructs in multiple small and large animal pre-clinical models. A rat skin fascial defect model was first implemented to assess an FDA-approved non-degradable polymer, polyamide (PA), which was warp-knitted to generate a mesh subsequently coated in cross-linked gelatin (PAG) for eMSC seeding and delivery [79]. The PAG meshes seeded with eMSC promoted more neovascularization and neo-tissue formation surrounding mesh filaments compared with unseeded PAG mesh. A more favourable response was also observed in the eMSC/PAG implanted tissue, where there was a switch to the M2 wound healing macrophage phenotype after early M1 inflammatory macrophage infiltration. Long-term chronic inflammation in the mesh–tissue complex was reduced in eMSC-seeded PAG mesh at 90 days, compared with PAG mesh alone [11,79]. Collagen fibres deposited around PAG eMSC-seeded mesh were crimped and more organized, improving the stiffness of the mesh/tissue complex and therefore the overall biomechanical properties of the tissue. However, eMSC only persisted for in-vivo for 2 weeks, emphasising that the more favourable tissue response was likely generated from paracrine anti-inflammatory effects that persisted long after the eMSC were reabsorbed [79]. A similar tissue engineered construct has been assessed in a large animal pre-clinical model, where PAG mesh was used either alone or seeded with autologous ovine eMSC. This study demonstrated that a simpler two step procedure of inserting uncoated polyamide (PA) mesh followed by the application of autologous eMSC in a gelatin hydrogel crosslinked in situ with blue light drastically improved tissue–mesh integration and resulted in no cases of mesh erosion [72].

### 4.2. eMSC Degradable Tissue Engineered Mesh 

**Figure 6:**
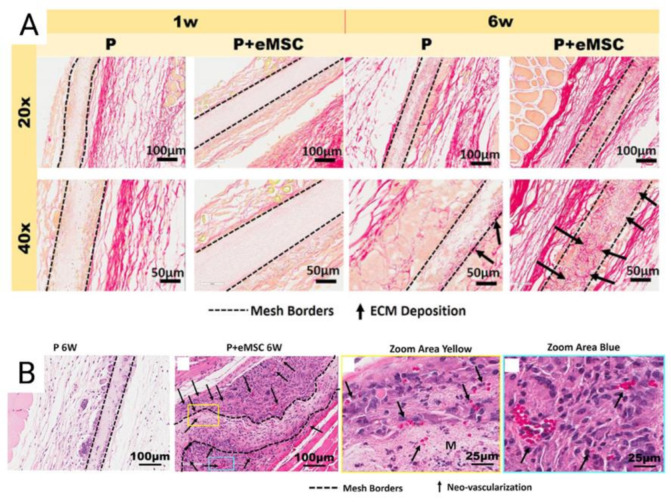
(**A**) Explanted Poly-L-lactide-co-ε-caprolactone (PLCL) nanomeshes with and without endometrial mesenchymal stem/stromal cells (eMSC) showing Picro-Sirius red staining of collagen, at 1 and 6 weeks explantation; (**B**) 6-week nanomesh explants with and without eMSC demonstrating improved neovascularisation (black arrows) within mesh area (yellow) with eMSC compared to without. Reprinted with permission from Mukherjee et al. (2020) [66]. Copyright: © 2021 by the authors.

Tissue engineering of degradable electrospun meshes with eMSC have demonstrated improvements in mesh integration. Electrospinning is an emerging versatile method of mesh construction that uses electric forces to draw charged threads of polymer solution together to create ultrafine meshes that allow for host immune cells to adhere in both nanofibrous and microfibrous formats [80,81]. These emerging biomimetic electrospun meshes produce an ECM-like topography that mimic the ECM of vaginal tissue of women with POP at the nanoscale [73]. Nanostructured meshes also provide a larger surface area for the delivery of eMSC and other adsorbing proteins and growth factors [19,73]. Poly-L-lactide-co-ε-caprolactone (PLCL) is a biocompatible, elastic, and flexible polymer investigated by several groups for novel tissue engineering applications, as its elastic modulus is well matched to the nanoarchitecture of vaginal tissue [81]. The properties of biomimetic PLCL were maximized by blending it with gelatin and incorporating eMSC in a mouse skin wound repair model. Blending of PLCL with gelatin forms nanostructured meshes and significantly increased the hydrophilicity and pore size of the meshes [73]. As a result, such blended meshes could be completely infiltrated by therapeutic eMSC despite a pore size of less than 3 μm. The presence of eMSC prevented rapid degradation of PLCL mesh, more effectively recruited host cells into the scaffolds, improved neovascularisation and successfully promoted an M2 macrophage phenotype to sustain a favourable inflammatory response [73]. Furthermore, eMSC seeded mesh resulted in an upregulation of genes controlling ECM deposition, cell adhesion and angiogenesis in tissues (Figure 6) [73].

In another study, PLCL meshes spun with fibrinogen (PLCL/Fg) have been shown to promote faster neo-vascularisation, better collagen fibre organisation, muscle regeneration, and no tissue erosion when compared with other polypropylene meshes, in a canine abdominal defect model [82]. This PLCL/Fg mesh was also trialled in human pelvic floor reconstruction (*n* = 38), with significant improvements in some aspects of POP-Q measurements when compared with the PP mesh group. Polylactic acid (PLA) meshes seeded with human and rat adipose-derived MSC implanted in a rodent abdominal wall defect model demonstrated improved macrophage infiltration, angiogenesis, ECM formation, and remodelling as indicated by increased deposition of collagen III [83]. PLCL meshes combined with eMSC have also demonstrated significant downregulation of several inflammatory genes in a 6-week mouse model, including *Tnf*, *Il1b*, *Ccl4*, *Ccl5*, *Ccl7*, *Ccl12*, *Ccl19*, *Cxcl1*, *Cxcll-2*, *Ccr1,* and *Ccr7*, and upregulation of anti-inflammatory genes involved in the M2 macrophage response like *Arg1* and *Mrc1* [66]. Thus, nanofiber electrospun mesh combined with eMSC have demonstrated a more favourable biomimetic and immunomodulatory profile that has the potential for applications in POP.

**Figure 7:**
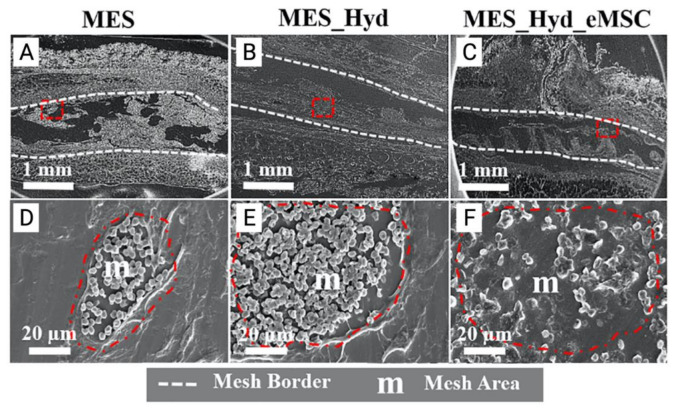
(**A**–**C**) 3D printed polycaprolactone (PCL) mesh explants visualised by electron microscopy one week after implantation. (**D**–**E**) Cross section of melt electrospun mesh (MES), MES_ Hydrogel and MES_Hydrogel_eMSC constructs (red dotted area) demonstrating improved integration with host tissue and neo-ECM formation (**F**). Reprinted with permission from Paul et al. (2019) [74]. Copyright Elsevier, 2019.

**Figure 8:**
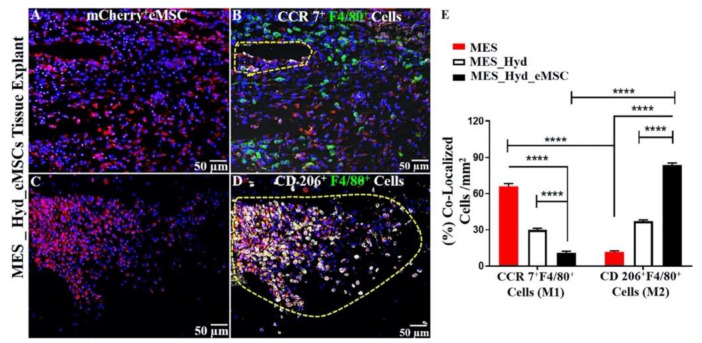
3D printed polycaprolactone (PCL) explants from NSG mice implanted for 1 week. (**A**,**C**) Demonstration of m-cherry^+^ eMSC retention; (**B**) recruitment of CCR7^+^ F4/80^+^ M1 Macrophages; (**D**) recruitment of CD 206^+^F4/80^+^M2 Macrophages (yellow cells in enclosed area); and (**E**) quantification of co-localized M2/M1 Macrophages showing relative predominance of CD 206^+^M2 macrophages (****; *p* < 0.0001). Reprinted with permission from Paul et al. (2019) [74]. Copyright Elsevier, 2019.

In a recent landmark pre-clinical study, our team developed the first known 3D-bioprinted polycaprolactone (PCL) mesh and evaluated its performance in vitro and in vivo. This mesh was constructed by a two-step process involving melt electrospinning (MES) followed by 3D bioprinting of therapeutic mCherry-labelled SUSD2^+^ eMSC in an aloe-vera-alginate hydrogel, and subsequently implanted under the skin of NOD scid gamma (NSG) mice. In vivo, acute foreign body response assessment revealed that eMSC bioprinted on electrospun mesh improved host tissue integration, eMSC retention, and promoted an anti-inflammatory M2 macrophage phenotype characterised by F4/80^+^CD206^+^ colocalization (Figure 7 and Figure 8) [74]. 

A novel aloe vera (AV)–alginate (ALG) hydrogel engineered to incorporate therapeutic SUSD2^+^ eMSC was used to effectively deliver cells following a simulated birth injury in a rat model [76]. In untreated subjects, vaginal trauma resulted in fibrotic healing, with a significant reduction in smooth muscle content and increased elastin, which increased tissue stiffness. Mice receiving AV-ALG hydrogel with eMSC, demonstrated a more favourable inflammatory response with a lower M1:M2 ratio. This resulted in reversal of fibrosis and restoration of vaginal connective tissue structure, with increased organised collagen deposition and reduced overall tissue stiffness [74]. Further studies are exploring the use of AV-ALG hydrogel in a large pre-clinical ovine animal model of birth injury, to optimize therapeutic cell delivery to vaginal tissue immediately after the simulated injury. This hydrogel-loaded MSC presents great potential as an immediate treatment for vaginal birth injury representing the first ever secondary preventative therapy for the development of POP. 

Irrespective of the method of biomaterial fabrication, it is imperative that new-generation meshes and hydrogels incorporate and deliver therapeutic cells such as eMSC that activate a favourable host immune response, rather than maladaptive chronic immune responses as seen with previous synthetic meshes [84,85]. To avoid mesh erosion, novel surgical constructs must have the desirable fibre alignment, stiffness, porosity, and topography to interact well with host vaginal tissue. Thus, personalised bioengineered constructs that are congruent with the topography of host vaginal tissue, and combined with therapeutic stem/progenitor cells, will promote the integration of mesh with native tissue. At the micro- and nanoscale level, cellular therapy has the potential to aid neovascularisation and deposition of neo-tissue that is more immunologically competent, resulting in a favourable surgical outcome for POP [86,87]. 

### 4.3. Large Pre-Clinical Animal Models of POP

Biomaterials combining eMSC with nanofiber meshes may be of great promise for improving POP surgery, though they have mostly been analysed in xenogenic rodent models of skin wound repair or abdominal hernia [39]. To avoid the complications associated with the rapid translation of non-degradable abdominal hernia mesh to transvaginal POP surgery under the 510(k) pathway, preclinical studies using large animal models is an imperative step in developing novel tissue engineered therapies. The domestic ewe has proven a good large animal model to perform pre-clinical studies in pelvic reconstructive surgery [88]. Anatomical, biomechanical, biochemical, and histological studies in nulliparous, primiparous, and multiparous ewes demonstrated similar rates of POP after vaginal delivery to that observed in humans, due to a large foetal head to body ratio and relatively comparable pelvic anatomy [88,89]. The diameter and length of the ovine and human vagina are relatively similar, and the ovine pelvic architecture relies on three levels of support, similar to the DeLancey model described in humans [90,91]. Both ovine and human vaginal wall consist of four histological zones of epithelium, lamina propria, muscularis, and adventitia [92]. Nulliparous, primiparous, and multiparous ewes demonstrated that parity results in greater displacements of the vaginal wall when using a modified POP-quantification (POP-Q) system adapted for sheep [93], which histologically correlated with an increased elastin and lower collagen content of vaginal wall, and thinning of the muscularis layer [89]. The modified POP-Q system has been adapted from the human POP-Q system, to assesses vaginal wall laxity at distinct anatomical landmarks in sheep, thereby providing an objective and reproducible measure of POP in anterior, posterior, and vault compartments. In this method, tissue forceps are used to assess maximum displacement of tissue at a reference point 3 cm proximal to the introitus at three possible points: Aa, 3 cm above the introitus on the anterior vaginal wall (range −3 above to +3 cm); Ap, 3 cm above the introitus (posterior wall); and Ba, above the urethra (anterior wall) (−3 cm to total vaginal length). Additional measurements include the length of the genital hiatus (GH), which is the vaginal orifice anterior to posterior (cm) and perineal body (PB), the distance from the middle of the anus to the edge of vaginal introitus with tail pulled back [93], (see Supplementary Figure S1). Cervical descent is not measured in the modified POP-Q as ewes have very long and relatively narrow vaginas (12–13 cm), with no obvious cervical descent [93]. 

**Figure 9:**
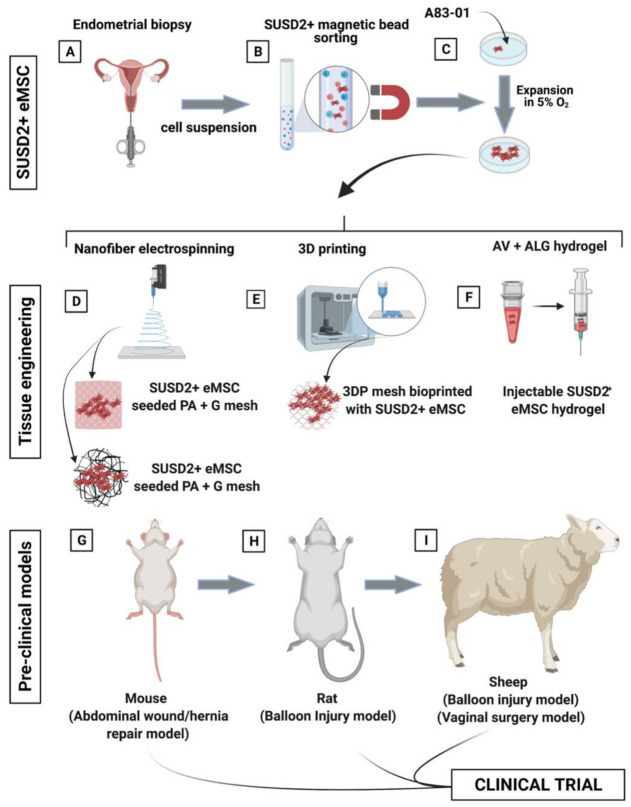
Schematic showing the process of creating tissue engineering constructs comprising eMSC- seeded degradable poly(L-lactic acid)-co-poly(e-caprolactone (PLACL) nanofiber electrospun mesh. (**A**) eMSC are obtained from endometrial biopsy specimens in an office-based procedure, (**B**) purified by SUSD2 magnetic bead sorting; (**C**) and expanded in serum free media containing A83-01 in 5% O_2_. (**D**–**F**) Generation of biomimetic tissue engineered constructs included nanofiber electrospinning, 3D printing, and an aloe vera–alginate (AV-ALG) injectable hydrogel; and (**G**–**I**) pre-clinical models including a murine wound or abdominal defect repaired with mesh, rat balloon injury for eMSC hydrogel injection, and a sheep balloon injury and vaginal surgery model for eMSC hydrogel injection and bioengineered nanomesh insertion, respectively.

Tissue engineered mesh inserted transvaginally in large animal models will aid the validation of these constructs prior to clinical translation by assessing their integration with host tissue and foreign body response through histological analysis, immunoassays and gene-profiling. In addition, the surgical and biomechanical efficacy of engineered mesh can be assessed at the nano- and macro-scale by implementing the modified POP-Q system, assessing tissue tensile strength testing, and collagen content analysis [74,76,89,93]. Research has commenced with the completion of multiple xenogenic small and large animal studies assessing eMSC/PAG constructs. Based on these findings, multiple heterologous small and large animal studies are underway to assess the efficacy of other biomimetic degradable materials such as PLCL and 3D PCL meshes seeded with eMSC (Figure 9). These animal models will be crucial in further assessing the efficacy of locally delivered eMSC and further determine their mechanism of action. 

## 5. Potential Clinical Applications of eMSC 

Various MSC-based therapies have performed poorly in clinical translation due to challenges in long-term engraftment of allogeneic MSC. Since perivascular eMSC and menstrual blood MSC derive from cyclically regenerating tissue, they present potential applications for tissue repair and possibly regeneration [40]. The ability to purify and characterise these cells through relatively non-invasive procedures has encouraged a separate investigation into their potential therapeutic applications in various clinical conditions with significant burden of disease [60]. As described, the restorative and regenerative capacity of autologous and heterologous perivascular eMSC is under investigation for applications in POP [8,11,19,39,44,72,73,74,76,79,80,81]. In particular, autologous ovine eMSC have persisted 30 days in a non-degradable PA mesh seeded with eMSC in situ in a transvaginal POP surgery model [72], xenogenic human eMSC for 1–2 weeks when implanted in a non-degradable PA+G composite mesh in immunocompromised rodent models of subcutaneous wound repair, and for 3 days in immunocompetent mice [70]. The eMSC exert strong paracrine effects which promotes early neovascularization, deposition of new collagen, and an improved anti-inflammatory response which is correlated with a reduction in stiffness of the mesh/tissue complex at 90 days [11]. In studies on PLCL/gelatin and 3D printed PCL constructs, eMSC also delayed mesh degradation, reduced the foreign body response, and induced endogenous cell influx to promote tissue repair [73,74]. These bioengineered constructs augmented with perivascular eMSC have the potential to improve host tissue response and ultimately prevent mesh exposure [60]. 

Menstrual blood MSC (MenSC), comprising unpurified endometrial stromal fibroblasts and eMSC derived from the shedding endometrium, have also been investigated as an attractive cell source for regenerative medicine [60]. These studies have demonstrated regenerative capacity in a myocardial infarct nude rat model, where they differentiated into striated, troponin I-expressing cardiac myocytes and reduced the infarcted area more than bone marrow MSC, which translated to an improvement in echocardiographic parameters of myocardial function [94]. Similar findings were demonstrated when MenSC were directly injected into ischaemic zones of an immunocompetent rat model of myocardial infarction, resulting in improved preservation of cardiomyocytes in the infarct zone [95]. MenSC have also demonstrated hepato-reparative potential in murine models of hepatic failure through paracrine effects that modulate liver fibrosis and by potentially differentiating toward hepatic cells [96,97,98]. In rodent models of ovarian insufficiency, MenSCs reduced apoptosis and increased numbers of primordial follicles which correlated with increased levels of serum anti-Mullerian hormone (AMH), oestrogen, and progesterone [99,100]. In a recent clinical trial in 15 women, intraovarian injection of autologous MenSC in poor ovarian responders increased clinical pregnancy and live births [101]. MenSC have demonstrated potential reparative application in other animal models of lung injury [102], wound repair [103], stroke [104], and muscular dystrophy [105]. 

In summary, perivascular eMSC and MenSCs have demonstrated promise in regenerative medicine through paracrine immunomodulatory effects and by enhancing endogenous stem cell function. The therapeutic potential of eMSC is further benefited by their relative ease of sampling, potency in culture, and reduced rates of spontaneous differentiation to fibroblasts under defined culture conditions [61]. Though hundreds of preclinical and a smaller number of clinical trials are underway, there remains much to be discovered about how the therapeutic potential of eMSC and MenSC can be harnessed for advancements in tissue repair and regenerative medicine. 

## 6. Limitations

Prior to translating the use of MSC to the clinic, their limitations and potential adverse effects must be thoroughly explored. Perivascular MSC are rare cells that are difficult to harvest from adult tissues, which necessitates substantial ex-vivo culture expansion in order to achieve sufficient numbers of potent cells. However, prolonged culture of MSC results in spontaneous differentiation to fibroblasts, an occurrence which significantly limits culture expansion. However, novel small molecule inhibitors can overcome this limitation, e.g., A83-01. Short-term engraftment of MSC demonstrated in multiple pre-clinical studies is particularly challenging, as their efficacy may be limited by the small proportion (2–10%) of therapeutic cells that are estimated to remain in vivo in the days following administration. The overactive host innate immune system in diseased or ischaemic tissue has been purported to rapidly remove allogenic MSC due to a loss of vascular niches that would otherwise aid tissue integration [106]. Despite these challenges, MSC exhibit important paracrine actions, appearing to reset the innate immune system and promote endogenous cellular repair despite their lack of tissue integration. Thus, a protective delivery system (e.g., hydrogel) that improves tissue retention of MSC to further augment therapeutic efficacy in tissues is imperative to ensure their success in clinical translation.

## 7. Summary and Future Perspectives

From a clinical perspective, there is an opportunity to explore personalised surgical approaches to POP where tissue engineered meshes are combined with therapeutic and potentially autologous eMSC. The severity and consequences of POP is highly related to individual patient’s anatomy, a variable that is often assessed pre-operatively with diagnostic radiology assessments through modalities such as endovaginal ultrasound (EVUS), 2D perineal pelvic floor ultrasound (pPFUS), transperineal ultrasound (TPUS), and translabial ultrasound (TLUS), and pelvic CT and MRI [107,108]. 

Pre-operative imaging studies can be integrated with computer-aided design (CAD) to produce a customised and patient-specific construct that is more likely to integrate with the unique dynamics of their anatomy and potential tissue defects. 3D bioprinting can thus be implemented to incorporate eMSC in a biocompatible hydrogel carrier that encapsulates cells and prints them layer by layer to construct a 3D functional living tissue, or artificial organ [109,110,111,112]. 

The key to the discovery of a safe and surgically efficacious tissue engineered mesh lies in a controlled design that highly considers the host tissue which it is intended. Tissue engineering joins the forces of nanotechnology, 3D printing, and eMSC to design a construct that addresses mesh–tissue mechanical mismatch, induces favourable tissue responses, and crucially mimics vaginal ECM topography. The highly regenerative human endometrium provides an exciting new, readily available, biologically active, and cost-effective source of MSC. Since their discovery, great advances have improved our ability to prospectively isolate these cells and maintain their clonogenicity during culture expansion, allowing their successfully implementation in a novel tissue engineering techniques. Thus, the use of eMSC in various homologous, autologous, and allogenic cellular therapies serve immense potential in advancing tissue engineering applications in POP, to alleviate a great clinical burden of disease.

## Figures and Tables

**Table 1:** Summary of endometrial MSC (eMSC)/stromal cell discoveries combined with novel tissue engineered scaffolds for applications in pelvic organ prolapse (POP).

Source/Model	Cell Type/Scaffold	Properties	Reference
Human	Freshly isolated stromal cells	Clonogenic, 1.25% cloning efficiency at seeding density (300/cm^2^). Fibroblastic and myofibroblastic morphology. Stromal colonies expressed CD90, 5B5.	Chan et al., 2004 [52]
Human	Freshly isolated CD146^+^PDGFRβ^+^(CD140b^+^CD146^+^) stromal cells	Enriched for colony-forming cells compared with CD146^−^PDGF-Rβ^−^ cells. Differentiation into adipogenic, osteogenic, myogenic, and chondrogenic lineages. Express ISCT * MSC surface markers. Pericyte locale.	Schwab et al., 2007 [55]
Human	SUSD2^+^ (W5C5^+^) eMSC	Single marker to isolate eMSC from endometrial cell suspensions using magnetic bead sorting. Differentiation into angiogenic, adipogenic, osteogenic, and myogenic lineages. Express lineage markers: CD90, CD146 and negative for CD31, CD34.	Masuda et al., 2012 [58]
Human	SUSD2^+^ eMSC/Polyamide (PA) nanomesh cross-linked with gelatin (PAG)	Gelatin cross-linking ↑ eMSC/PAG adherence and growth rate. Connective tissue growth factor induced eMSC differentiation into SM22a^−^ and SM-MHC-expressing smooth muscle cells. Induction of COL-I and Tn-C-expressing fibroblasult-like cells.	Su et al., 2014 [67]
Human/Rat (Subcutaneous wound)	SUSD2^+^ eMSC/Polyamide (PA) nanomesh cross-linked with gelatin (PAG)	Compared to mesh without eMSC: ↑ neovascularization and neo-tissue formation surrounding mesh filaments versus. unseeded PAG mesh. Early M1 macrophage infiltration followed by ↑ M2 macrophage switching. ↓ macrophages around mesh filaments and ↓ mesh–tissue complex stiffness at 90 days. Deposition of physiological crimped collagen. ↑ neovascularization and ↓ leukocyte infiltation at 7 days. Survival of DIO labelled eMSC up to 14 days postimplantaiton.	Ulrich et al., 2014 [11] Edwards et al., 2015 [68]
Human	SUSD2^+^ eMSC	TGFβR inhibitor, A83-01 maintains SUSD2^+^ eMSC stemness during culture expansion in serum free medium. A83-01 dose-dependently ↑ SUSD2^+^ cell proliferation, and ↓ cell apoptosis and senescence. Express CD90, CD140b, CD146, but not CD271.	Gurung et al., 2015 [61]
Sheep	CD271^+^CD45f^−^ ovine eMSC	↑ cloning efficiency and serial cloning capacity than CD271^−^CD49f^−^ cells. Differentiation into adipocytic, smooth muscle, chondrocytic, and osteoblastic lineages. Adventitial perivascular location.	Letouzey et al., 2015 [69]
Human/NSG and C57Bl6 mice (wound repair)	mCherry^+^ SUSD2^+^ eMSC/PAG mesh	↑ persistence in immunocompromised compared to immunocompetant mice. ↓ inflammatory cytokine production (Il-1β, Tnfα) versus. PAG mesh without eMSC. ↓ CCR7^+^ M1 macrophages surrounding mesh on day 3 in C57BL6 mice. ↑ M2 macrophage response with ↑ mRNA expression (*Arg1*, *Mrc1*, *Il10)* at 3 and 7 day in C57BL6 mice.	Darzi et al., 2018 [70]
Human/NSG mice	mCherry^−^labelledSUSD2^+^ eMSC	Lentivirus mCherry transduction effectively labels SUSD2^+^ eMSC with maintenance of mCherry expression over five passages. mCherry^+^ eMSC encapsulated in fibrin gel and transplanted under the subrenal capsule detectable in vivo at 30 days.	Gurung et al., 2018 [71]
Human	A83-01 treated SUSD2^+^ eMSC	Induction of anti-fibrotic genes: *HGF*, *CCL2*, *FBLN1*, *VWA1*, *TGFB3*, and *MIR155HG*. Induction of anti-apoptotic genes: *FAIM2*, *NFKBIA*, *ENPP2*, *SEPTA*. ↑ expression of bone marrow MSC potency genes: *TWIST1*, *TWIST2*, *JAG1*, *LIFR*, and *SLIT2*. MSC exosome marker expression: *CD63*, *CD9*, *CD81*, and *CD82*. ↓ TGF-β, Wnt and Akt signalling pathways. Genetic stability of of telomeres through upregulation of *TERC TERF1/2*, *TINF2*, *TERF2IP*, *TNKS*, and *POT1*.	Gurung et al., 2018 [62]
Sheep	Autologous ovine CD271^+^ eMSC/PAG mesh	PA mesh + eMSC/G improved tissue–mesh integration. ↓ myofibroblast and inflammatory response. ↓ muscularis layer disruption. ↓ elastin. No cases of mesh erosion. Autologous ovine eMSC persisted for 30 days in ovine vagina.	Emmerson et al., 2019 [72]
Human/NSG mice (wound repair)	SUSD2^+^ eMSC/PLCL nanomesh	↑ neovascularization and M2 macrophage response. ↓ expression of inflammatory genes at 6 weeks, *Tnf*, *Il1b*, *Ccl4*, *Ccl5*, *Ccl7*, *Ccl12*, *Ccl19*, *Cxcl1*, *Cxcll-2*, *Ccr1,* and *Ccr7*. ↑ expression of anti-inflammatory genes *Arg1* and *Mrc1*. Induction of CD206^+^ multinucleated foreign body giant cells with smaller nuclei. ↑ ECM synthesis and expression of cell adhesion markers.	Mukherjee et al., 2019 [73] Mukherjee et al., 2020 [66]
Human/NSG mice (wound repair)	mCherry-labelled SUSD2^+^ eMSC/3D on 3D printed PCL nanomesh	bioprinting in aloe-vera-alginate bioink improved host tissue integration and eMSC retention. ↑ anti-inflammatory CD206^+^F4/80^+^ M2 macrophages ↓ CCR7^+^ F4/80^+^ M1 macrophages.	Paul et al., 2019 [74]
Human	A83-01 treated SUSD2^+^ eMSC	After 5 weeks ↑ cell growth gene expression, ↓ extracellular matrix and cell fate genes. Opening and closure of 3555 and 2412 chromatin loci, respectively revealed by ATAC-seq. Motif analysis of open chromatin regions revealed, ↑ induction of retinoic acid receptor beta (RARβ) and upgregulation of RA gene signalling genes (RNAseq). Selective RARβ inhibition ↓ eMSC proliferation and clonogenicity.	Lucciola et al., 2020 [63]
Human	A83-01-treated SUSD2^+^	↑ eMSC proliferation cultured from post-menopausal endometrium, menstrual blood, and placenta decidua-basalis. ↑ cloning efficiency ↑ SUSD2+ expression to 85–95% of cultured cells ↓ senescence and apoptosis. A83-01 had no effect on bone marrow MSC proliferation and adipose MSC senescence.	Gurung et al., 2020 [75]
Human/rat (vaginal birth injury)	SUSD2^+^ eMSC/AV+ALG hydrogel	Effective delivery of SUSD2^+^ eMSC in AV+ALG injectable therapy ↓ fibrotic healing, ↓ smooth muscle loss, ↓ elastin, ↓ nanoscopic tissue stiffness Compared with AV+ALG alone improved immune response with ↓ M1:M2 ratio.	Paul et al., 2021 [76]

* ISCT, International Society for Cellular Therapy.

## Supplementary Materials

The following are available online at https://www.mdpi.com/article/10.3390/jpm11090840/s1, Figure S1: (A) Modified Pelvic Organ Prolapse Quantification System (POP-Q): definition and locations in the ewe. *Ap* 3 cm above the introitus in the posterior vaginal wall (range −3 above to +3 cm). *Aa* 3 cm above the introitus on the anterior vaginal wall (range −3 above to +3 cm). *Ba* above the urethra (anterior wall) (−3 to total vaginal length). (B) Traction applied with tissue forceps at Ap, Ba and Aa, at points as marked. Ewes with displacement +1 were considered to have POP. Copyright (Young et al.), reproduced with permission [93]. GH, genital hiatus; PB, perineal body; TVL, total vaginal length.
