# Supplementary figures and images for: Endometrial SUSD2+ Mesenchymal Stem/Stromal Cells in Tissue Engineering: Advances in Novel Cellular Constructs for Pelvic Organ Prolapse
Source: J Pers Med. 2021 Aug 26;11(9):840. doi: 10.3390/jpm11090840 (PMC8471527; doi:10.3390/jpm11090840)

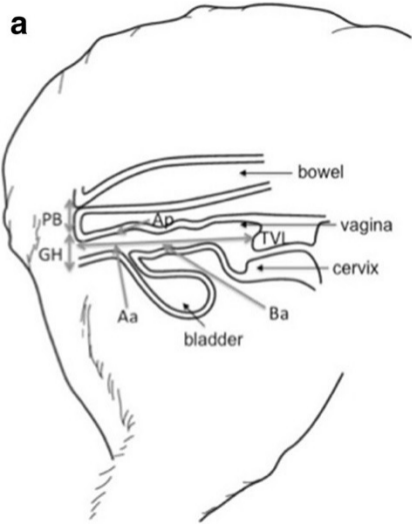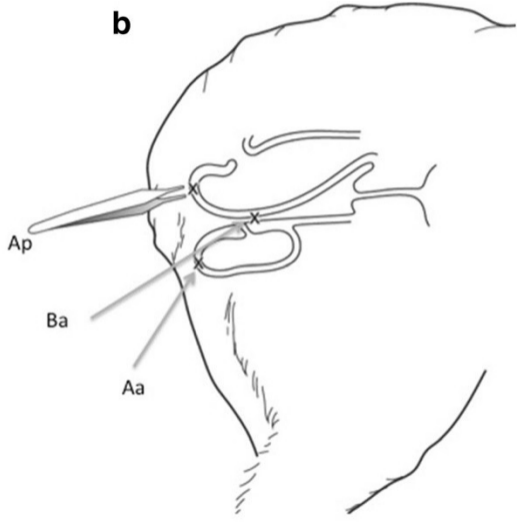

Supplement: Supplementary file 1 [file jpm-11-00840-s001.zip › jpm-1315325-supplementary.pdf]
